# Bioconcentration of carbamazepine, enalapril, and sildenafil in neotropical fish species

**DOI:** 10.3389/ftox.2023.1247453

**Published:** 2023-10-03

**Authors:** Macarena Gisele Rojo, Diego Cristos, Pedro Carriquiriborde

**Affiliations:** ^1^ Centro de Investigaciones del Medio Ambiente (CIM), Facultad de Ciencias Exactas, Universidad Nacional de La Plata-CONICET, Buenos Aires, Argentina; ^2^ Centro de Investigaciones de Agroindustria, Instituto Nacional de Tecnología Agropecuaria, (CIA-INTA), Buenos Aires, Argentina

**Keywords:** pharmaceuticals, bioconcentration, fish, Del Plata Basin, bioconcentration kinetics

## Abstract

Sewage effluents are the main source of entry of Human Pharmaceutical Active Ingredients (HPAIs) to surface water bodies. Carbamazepine (CBZ), psychiatric drug, enalapril (ENA) antihypertensive, and sildenafil (SIL), to treat erectile dysfunction, have been frequently detected in receiving wastewater and in wild fish species from Argentina. This study aimed to assess the bioconcentration of selected HPAIs in native fish species of the Del Plata Basin. In a first trial, the bioconcentration factors of CBZ, ENA, and SIL were obtained by exposing *Cnesterodon decemmaculatus*, respectively, to 135, 309, and 70 μg/L during 96 h. Then the bioconcentration kinetic of SIL was comparatively assessed in *C. decemmaculatus* and *Piaractus mesopotamicus* exposed, respectively, to 44.1 and 16.2 μg/L during a one-week, followed by a four-day depuration phase. HPAIs concentrations in water and tissue were measured by HPLC-MS after 0.22 μm filtration and direct injection or solid-liquid extraction, respectively. Bioconcentration factors obtained empirically (BCF_e_) for *C. decemmaculatus* were CBZ = 1.5, SIL = 1.4, and ENA = 0.007. Parameters estimated by the SIL bioconcentration kinetic model for *C. decemmaculatus* were: uptake rate constant (k_1_) = 5.5 L/kg d, elimination rate constant during uptake phase (k_2u_) = 0.00175 d^−1^, maximum predicted tissue concentration (C_t(max)_) = 138588 μg/kg, estimated bioconcentration factor (BCF_m_) = 3143, lag time between the exposure and the first detection in tissue (t_lag_) = 0 d, elimination rate constant in the depuration phase (k_2d_) = 0.49 d^−1^ and half-life in the tissue (t_1/2_) = 1.4 d. The model parameters for *P. mesopotamicus* were k_1_: 7.3 L/kg d, k_2u_: 0.0836 d^−1^, C_t(max)_: 1423 μg/kg, BCF_m_: 88, t_lag_: 3.8 d in the uptake phase and k_2d_: 0.31 d^−1^ and t_1/2_: 2.3 d in the depuration phase. The reached conclusions were: 1) the bioconcentration capacity of CBZ and SIL are similar but around 200 times higher than ENA, 2) the time to reach the bioconcentration equilibrium for SIL is longer than 1 week, then estimated BCF_m_ are between 1 and 3 orders of magnitude higher than BCF_e_ obtained after 96 h exposure, but actual values need to be verified, 3) substantial differences (≈30 fold) were observed in the estimated BCF of SIL among species, indicating the need for further studies toward understanding such diversity to improve HPAIs ecological risk assessment worldwide.

## 1 Introduction

Human pharmaceuticals active ingredients (HPAIs) are considered emerging pollutants, and manufacturing, hospitals, and domestic wastewater discharges are their relevant sources for aquatic ecosystems (Patel et al., 2019). In polluted environments, aquatic organisms are exposed to HPAIs through different pathways, including absorption from water and food (Corcoran et al., 2010). The study of the bioaccumulation of HPAIs in aquatic biota and the influencing factors have been considered a priority research topic in ecotoxicology (Boxall et al., 2012).

The occurrence of HPAIs in surface waters has been detected worldwide but detected compounds varied among regions, and low-to-middle-income countries presented higher concentrations (Wilkinson et al., 2022). In wastewaters and receiving waters of Argentina, atenolol, caffeine, carbamazepine (CBZ), diclofenac, enalapril (ENA), ibuprofen, and sildenafil (SIL) were those more frequent and usually showing concentrations above the μg/L (Elorriaga et al., 2013a; Elorriaga et al., 2013b; Pérez et al., 2018). Additionally, HPAIs were detected in the muscle of wild fish of the Del Plata Basin: *Pimelodus maculatus*, *Prochilodus lineatus*, and *Megaleporinus obtusidens*, with maximum concentrations of 71.6, 69.4, 56.9, and 45.8 μg/kg of SIL, ATE, ENA, and CBZ (Rojo et al., 2019; Rojo et al., 2021).

Laboratory-exposed fish studies have shown that HPAIs bioconcentration usually reaches the steady-state plateau before a week of exposure (Garcia et al., 2012; Wang and Gardinali, 2013; Brox et al., 2016). In Argentina, few laboratory trials have been conducted to evaluate the bioaccumulation of HPAIs in native fish. The study of Valdés et al. (2016) assessing the short-term (48 h) bioconcentration of the anti-epileptic CBZ in the local fish *Jenynsia multidentata* is one of the few currently available. Additionally, no studies were found evaluating the bioaccumulation of the antihypertensive ENA and the erectile dysfunction drug SIL in fish under laboratory conditions.

*Cnesterodon decemmaculatus* (Jenys, 1842) and *Piaractus mesopotamicus* (Holmberg, 1887) are Neotropical fish species characteristics of the Del Plata Basin (Lucinda, 2005; Calcagnotto and DeSalle, 2009). *C. decemmaculatus* is a small poeciliid abundant in vegetated streams of the southern sector of the Basin and commonly used in ecotoxicological studies (Carriquiriborde et al., 2007; Vera-Candioti et al., 2015; Young et al., 2020; Baudou et al., 2021). Female individual size can reach a maximum of 3.7 cm, and their weight range from 50.1 to 524.1 mg (Ferrari, 2017). *P. mesopotamicus* inhabits the northern sector of the Del Plata Basin (Paraná-Paraguay River). It is also used in ecotoxicological studies. Unlike *C. decemmaculatus*, *P. mesopotamicus* is important for local aquaculture and human consumption (Brodeur et al., 2021; Carrizo et al., 2022). Size can reach a maximum of 60 cm and weigh up to 8 kg (CARU, 2019; Brodeur et al., 2021).

This study aimed to compare the bioconcentration factors of CBZ, ENA, and SIL in *C. decemmaculatus* and to compare the bioconcentration kinetic of SIL in *C. decemmaculatus* and *P. mesopotamicus*.

## 2 Materials and methods

### 2.1 Standards and reagents

Carbamazepine (CBZ) CAS No 298-46-4, log Kow 2.77, pKa 2.77 and 13.9 (Loftsson et al., 2010), enalapril (ENA) CAS No 76095-16-4, log Kow 2.45, pKa 3.0 and 5.5 (Huerta et al., 2013a; Huerta et al., 2013b), and sildenafil (SIL) CAS No 171599-83-0, log Kow 3.18, pKa 6.78 and 9.12 (Gobry et al., 2000). High degree purity (90%) standards were from Parafarm^®^, Argentina. Analytical grade acetone and ethyl acetate were JT Baker^®^. Chromatographic mobile phase solvents were acetonitrile and HPLC-grade methanol was Carlo Erba. Ammonium formate (99%) and formic acid (88%) were Sigma-Aldrich.

### 2.2 Test organisms

Adult females of *C. decemmaculatus* were caught at the La Plata city park lake (Lago del Bosque), La Plata City, Buenos Aires, Argentina. Juvenile *P. mesopotamicus* were acquired from Teko Company, Province of Chaco, Argentina. Fish were maintained 2 weeks before the test in the aquaria of the Centro de Investigaciones del Medioambiente (CIM-CONICET-UNLP). The 2,000 L aquaria system was filled with dechlorinated and activated charcoal-filtered tap water at 24°C ± 2°C, pH 7.5 ± 0.5, and oxygen 8 ± 1.2 mg/L with 16:8 h light/dark cycle, constant aeration, and fish were fed twice daily based on a feed for omnivorous fish from Mixes del Sur. The weight and standard length of the fish at the start of the experiment were 0.3 g and 23 mm for *C. decemmaculatus* and 48 g and 100 mm for *P. mesopotamicus*. Fish were handled according to the protocol approved by the Commission for the Care and Use of Laboratory Animals of the Faculty of Exact Sciences of the National University of La Plata (CICUAL # 006-26-17).

### 2.3 Experimental design

Bioconcentration factor trials: thirty-six *C. decemmaculatus* adult females were randomly distributed (12 per tank in 3 tanks) and exposed to 200, 600, and 100 μg/L of CBZ, ENA, and SIL, respectively, for 96 h. The exposure concentrations were set based on the acute lethal toxicity (≈1/100 LC_50_) obtained for *C. decemmaculatus* in a preliminary test. Also, a control group was included using the same concentration of vehicle solvent [0.01% (v/v) methanol]. Exposure time was selected according to the bioconcentration kinetic found in the literature for most HPAIs and because it is a broadly used exposure time in fish ecotoxicity tests (Garcia et al., 2012; Wang and Gardinali, 2013; Brox et al., 2016).

Testing aquaria were 20 L stainless steel tanks filled with 12 L of dechlorinated La Plata tap water filtered through active charcoal (pH 7.5) and aerated for 48 h before the trial. The bioconcentration tests were performed at the same light and temperature conditions used for fish maintenance. Partial media renewal, 80% of the volume, was done at 48 h, just after fish feeding. Fish were sampled, killed in ice-cold water, and stored at −80 °C until processing. Composite samples were performed using the whole fish.

Bioconcentration kinetic trials with *C. decemmaculatus*: an 11-d test was conducted, including a 7 d uptake phase exposing fish to 50 μg SIL/L, followed by a 4 d depuration phase in which the test solution was replaced by dechlorinated and filtered water. Also, a control group with dechlorinated water and vehicle solvent [0.01% (v/v) methanol] was included. The tested concentration was based on the BCF obtained in the 96-h trial (1.4), and the concentration measured in fish collected in the field (71.7 μg/kg) in a former study (Rojo et al., 2021), a fact that estimates a concentration in the water of 51.2 μg/L. Each treatment was carried out in triplicate, placing four adult females in each of the eighteen 3 L glass aquaria filled with 2.5 L of test solution. Total renewal of the test solutions was carried out every 48 h, and food was only provided at the end of the uptake phase. Aquaria were randomly sampled at 0, 1, 3, 5, 7, 9, and 11 d. The four fish of the same aquaria were killed in ice-cold water, whole bodies pooled, wrapped in aluminum foil, placed in food-grade bags, and stored at −80 °C until processing. The test was performed under the same conditions used for fish maintenance.

Bioconcentration kinetic trial with *P. mesopotamicus*: a 10 d test was conducted, including a 6 d uptake phase exposing fish to 50 μg SIL/L, followed by a 4 d depuration phase, in which the test solution was renewed by dechlorinated water. Also, a control group with dechlorinated water and vehicle solvent [0.01% (v/v) methanol] was included. Each treatment was carried out in triplicate, placing three juveniles in each of the eighteen 20 L stainless steel aquaria filled with a volume of 10 L of test solution. Aquaria were randomly sampled at 0, 2, 4, 6, 8, and 10 d. Partial renewal of the test solution (80%) was done every 48 h, just after fish feeding. Fish were sampled, killed in ice-cold water, and dissected with stainless steel instruments. The dorsal muscles of both flanks were sampled, wrapped in aluminum foil, placed in food-grade bags, and stored at −80 °C. Muscle instead whole fish was used because it is an eatable fish. The test was performed under the same conditions used for fish maintenance.

### 2.4 Chemical analysis

Water samples from each trial were collected at the beginning and the end of the exposure period (96 h) and analyzed by direct injection after filtration through a 0.22 μm nylon filter. Extraction and analyses of samples were carried out following the analytical methodology by Ramirez et al. (2007) and adapted by Rojo et al. (2021). Samples (0.5 g) were homogenized in 4 mL of ethyl acetate-acetone (50:50) using a Bio-gen PRO200 homogenizer at 30,000 rpm. Then, homogenates were centrifuged at 1,000 *g* for 10 min at 4°C. Supernatants were transferred to 15 mL conical glass tubes and taken to dryness under a gentle nitrogen stream. Finally, extracts were resuspended in 500 µL of methanol-acetonitrile (50:50), transferred to a chromatographic vial, and stored at −20 °C.

Analysis of pharmaceuticals was done by LC-ESI-MS, using a Waters^®^ ultraperformance liquid chromatography system ACQUITY UPLC^®^ equipped with an autosampler and coupled to a single mass spectrometer ACQUITY SQD^®^. LC-MS instrumental settings have been detailed in Rojo et al. (2021). Briefly, chromatographic separation of pharmaceuticals was performed in an XSelect CSH C18 column (3.5 µm, 4.6 × 75 mm), using acetonitrile/methanol (50:50) and ultrapure water as mobile phase at a flow rate of 0.3 mL/min and injection volume of 20 µL. Used modifiers in both phases were formic acid (0.1%) and ammonium formate (5 mM). Mass spectrometer parameters were: capillary, and cone voltage 3240 V and 35 V, respectively, cone gas flow 1.1 mL/s, desolvation gas flow 193 mL/s, and source and desolvation temperature were 148°C and 350°C, respectively. Single ion monitoring (SIM) under positive ionization mode was applied to quantify and confirm selected ions for each pharmaceutical. Detailed ions and retention times are shown in Supplementary Table S1. Method detection limits (MDL) and method quantification limits (MQL) were expressed as 3 and 10 times the standard deviation of the reagent blank. To avoid matrix effects, quantification was performed using the Standard Addition Method (APHA-AWWA-WEF, 2017). A standard calibration curve with three points, 10, 100, and 1,000 μg/kg, was prepared in control tissue extracts of both fish species. Recovery percentages were verified by duplicate using the whole body homogenates of *C. decemmaculatus,* spiked at 100 μg/kg.

### 2.5 Data analysis

Statistical analysis was performed using dedicated software. The empirical bioconcentration factors (BCF_e_) were obtained as the ratio between the average concentration of the pharmaceutical in the tissue after 96 h exposure (C_t(96h)_) and the average concentration in the testing solution (C_ts(av)_) (Eq. 1). Concentrations in tissue were expressed as the average ± standard error (w.w) and average concentrations in the testing solutions (C_ts(av)_) were expressed as the average concentration in water during the test using the concentration in water at the start C_w(t0)_ and the end of the test C_w(96h)_ (Eq. 2).

$${BCF}_{e}=\frac{C_{t\left(96h\right)}\;\left(µg/kg\right)}{C_{ts\left(av\right)}\left(µg/L\right)}$$ (1)

$$C_{ts\left(av\right)}=\frac{C_{w\left(t0\right)}\left(µg/L\right)+C_{w\left(96h\right)}\;\left(µg/L\right)}{2}$$ (2)

Bioconcentration factors estimated by means of the kinetic model (BCF_m_) were calculated presuming first-order kinetics, as the ratio between the uptake rate constant (k_1_) and depuration rate constant of the uptake phase (k_2u_) (Eq. 3). Both parameters were estimated from the kinetic bioconcentration model (Eq. 4) (Newman, 2013). Adjusted to the empirical data using statistical software.

$${BCF}_{m}=\frac{k_{1}}{k_{2u}}$$ (3)

$$C_{t}=C_{ts\left(av\right)}\times \left(\frac{k_{1}}{k_{2u}}\right)\times \left(1-\exp^{\left(-\right.k_{2u}x\;\left(t-t_{lag}\right)}\right)$$ (4)

Where, C_t_ (µg/kg): is the pharmaceutical concentration in the tissue at time t, C_ts(av)_ (µg/L): is the pharmaceutical average concentration in the testing solution during the entire test, k_1_ (L/kg d): is the uptake rate constant, k_2u_ (d^−1^): elimination rate constant in the uptake phase, k_2d_ (d^−1^): elimination rate constant in the depuration phase, t (d): exposure time, t_lag_ (d) latency time (time delay between the exposure and the detection in tissue).

The model applied describes the accumulation through time that eventually approaches steady-state equilibrium with the source. The concentration in the fish at steady-state equilibrium is represented by C_t(max)_ (µg/kg), (Newman, 2013) (Eq. 5). Reaching the steady state (t_∞_), the term {1-exp [-k_2u_ x (t-t_lag_)]} of Eq. 4 takes the value 1.

$$C_{t\left(\max\right)}=C_{ts\left(av\right)}\times \left(\frac{k_{1}}{k_{2u}}\right)$$ (5)

The elimination rate constant of the depuration phase [k_2d_ (d^−1^)] was obtained based on Eq. 6.

$$C_{t}=\exp^{\left(-\right.k_{2d}\left.x\;t\right)}$$ (6)

Half-life (t_1/2_ (d)), the time required for the concentration of SIL to fall to 50% of its initial value, was calculated with Eq. 7.

$$t_{1/2}=\frac{\ln\left(0.5\right)}{k_{2d}}$$ (7)

## 3 Results

The experimental conditions were unchanged during all tests, and no mortality or significant weight gain was observed in fish at any trial. The detection and quantification limits for the pharmaceuticals determinations in the water ranged between 0.067 and 0.24 μg/L and 0.22 and 0.81 μg/L, respectively. The method recoveries for the internal concentrations analysis were 48, 106, and 72%, and detection and quantification limits were 0.14 and 0.46 μg/kg, 0.23 0.76 μg/kg, and 0.21 and 0.70 μg/kg for CBZ, ENA, and SIL, respectively.

### 3.1 Bioconcentration factors of CBZ, ENA, and SIL in *C. decemmaculatus*

The average concentrations of CBZ, ENA, and SIL in water during the tests and in whole fish after 96 h of exposure are shown in Table 1. Reductions of 80, 92, and 90% in water concentrations were observed for CBZ, ENA, and SIL between the 48 h renewal intervals (Supplementary Table S2). None of the studied pharmaceuticals were detected in the water samples of the control group. The concentrations in the fish were as maximum as 1.5-fold the ones in the water. Therefore, the calculated BCF_e_ values were relatively low for the three compounds. Moreover, while the BCF_e_ values for CBZ and SIL were similar and close to one, the value for ENA was two orders of magnitude smaller.

**TABLE 1:** Empirically obtained bioconcentration factors (BCF_e_) for CBZ, ENA, and SIL in *C. decemmaculatus*.

Compound	C_ts(av)_ ± SE	C_t 96 h_ ± SE	BCF_e_
Carbamazepine	135 ± 9.08	203 ± 2.89	1.5
Enalapril	309 ± 2.63	2.16 ± 1.86	0.007
Sildenafil	70.4 ± 5.78	102 ± 7.31	1.4

C_ts(av):_ average concentration in the test solution (µg/L); C_t 96h:_ average concentration in tissue (whole fish) after 96 h of exposure (µg/kg); SE: standard error (n = 3); BCF_e_: Empirically Bioconcentration Factor.

### 3.2 Bioconcentration kinetic of SIL in *C. decemmaculatus*

**FIGURE 1:**
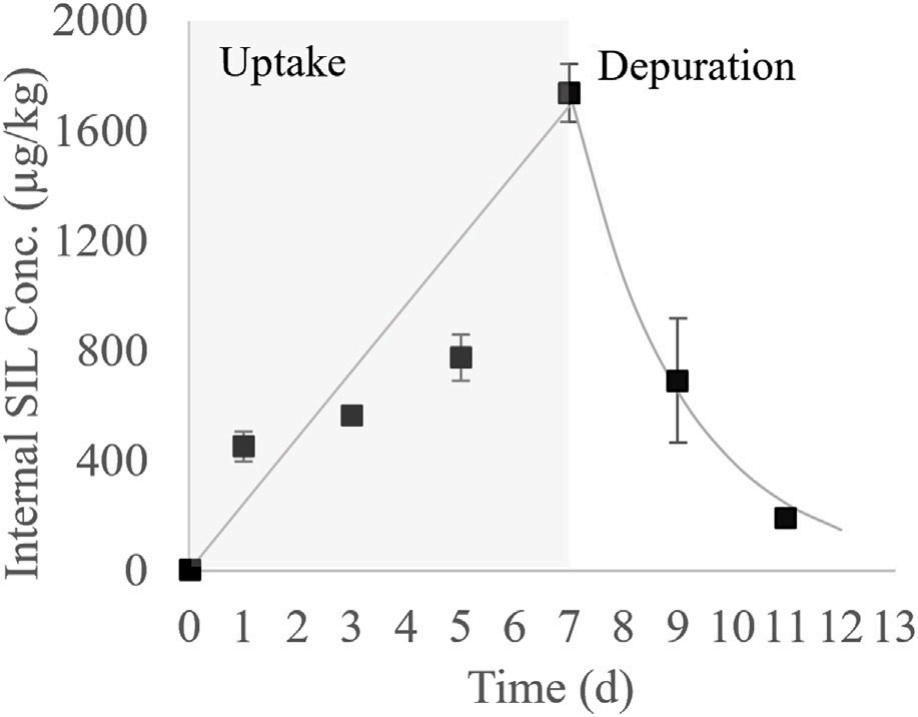
Bioconcentration kinetic of SIL in *C. decemmaculatus* (black squares: average measured concentration ± the standard error; solid line: first order bioconcentration model fitted to the uptake and depuration phases).

The average concentration of SIL in water during the *C. decemmaculatus* test was 44.1 ± 4.02 μg/L, again showing a marked decay (98%) between renewals. Internal concentrations of SIL in the whole *C. decemmaculatus* are shown in Supplementary Table S3. In Figure 1, these concentrations were plotted as a function of the time, showing a quick and significant accumulation since the first day of exposure, followed by a sustained increase until the end of the exposure phase. The maximum internal concentration of SIL was 1769 μg/kg on day seven, which equated to a BCF_e_ of 40. Quick drug clearance was observed during the 2 days depuration phase, showing only 13% of the maximum accumulated concentration on day 11.

The first-order bioconcentration kinetic model fitted to empirical internal concentrations during the uptake and depuration phases is also shown in Figure 1. The respective parameters are shown in Table 2. The uptake phase was characterized by the linear term of Eq. 4, indicating that during the tested time the SIL elimination velocity was negligible. Marked differences between elimination constants were estimated for the uptake and depuration phases, being the k_2u_ 280-fold smaller than k_2d_. The estimated values for the maximum concentration at the plateau and the BCF_m_ were comparatively high due to the small k_2u_ value. No lag time was predicted in the uptake phase by the model. Additionally, the depuration half-time was estimated in less than 2 days.

**TABLE 2:** First-order bioconcentration kinetic model parameters for SIL accumulation in *C. decemmaculatus*.

Uptake phase						Depuration phase		
k_1_	k_2u_	C_t max_	BCF_m_	t_lag_	*r* ^2^	k_2d_	t_1/2_	*r* ^2^
5.5	0.00175	138,588	3,143	0	0.87	0.49	1.4	1.0

k_1_: uptake rate constant (L/kg d), k_2u_: depuration rate constant of uptake phase (d^−1^), C_t max_: maximum concentration in whole body estimated by the model (µg/kg), BCF_m_: bioconcentration factor estimated by the kinetic model (L/kg), t_lag_: latency time (d), k_2d_: depuration rate constant of depuration phase (d^−1^), t_1/2_: half-life (d), *r* ^2^: correlation coefficient.

### 3.3 Bioconcentration kinetic of SIL in *P. mesopotamicus*

**FIGURE 2:**
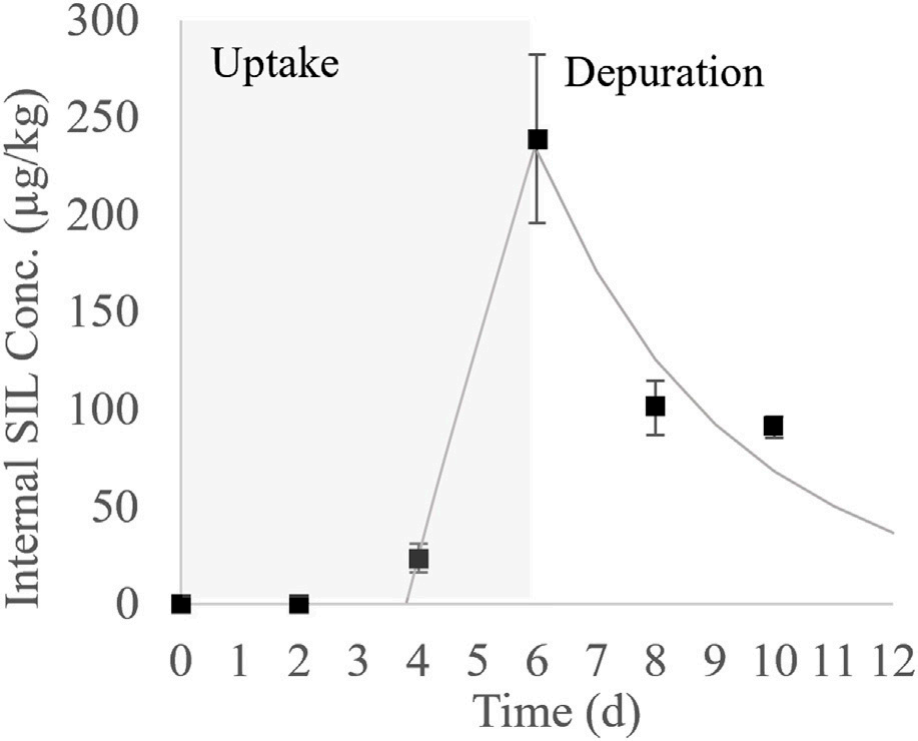
Bioconcentration kinetic of SIL in *P. mesopotamicus* (black squares: average measured concentration ± the standard error; solid line: first order bioconcentration model fitted to the uptake and depuration phases).

The average concentration of SIL in the water during the *P. mesopotamicus* test was 16.2 μg/L, dropping by 99% between renewals. Internal concentrations of SIL measured in *P. mesopotamicus* during the uptake and depuration phase are shown in Supplementary Table S4. In Figure 2, these concentrations were plotted as a function of the time, showing a lag time of 4 days before the concentrations were detected in the muscle. Then, a significant accumulation was observed until the end of the uptake phase, reaching a maximum internal concentration of SIL of 241 μg/kg on day six. The calculated BCF_e_ at that point was 15. During the four-day depuration phase, 60% of the maximum concentration was eliminated.

The first-order bioconcentration kinetic model fitted to the empirical internal concentrations during the uptake and depuration phases is also plotted in Figure 2. The respective parameters are shown in Table 3. The uptake phase was characterized by a 3.8 d lag time followed by a linear increment of the tissue concentrations, the last, indicating that SIL elimination velocity was negligible during this period. Differences between elimination constants were estimated for the uptake and depuration phases, with the k_2u_ 3.70-fold smaller than k_2d_. The estimated values for the maximum concentration at the plateau and the BCF_m_ were higher than measured from empirical data at the end of the uptake phase. The depuration half-time was quick, estimated at around 2 days.

**TABLE 3:** First-order bioconcentration kinetic model parameters for SIL accumulation in *P. mesopotamicus*.

Uptake phase						Depuration phase		
k_1_	k_2u_	C_tmax_	BCF_m_	t_lag_	*r* ^2^	k_2d_	t_1/2_	*r* ^2^
7.3	0.0836	1,423	88	3.8	1.0	0.31	2.3	0.92

k_1_: uptake rate constant (L/kg d), k_2u_: depuration rate constant of uptake phase (d^−1^), C_t max_: maximum concentration in whole body estimated by the model (µg/kg), BCF_m_: bioconcentration factor estimated by the kinetic model (L/kg), t_lag_: latency time (d), k_2d_: depuration rate constant of depuration phase (d^−1^), t_1/2_: half-life (d), *r* ^2^: correlation coefficient.

## 4 Discussion

Analytical methods performance was according to previous studies (Rojo et al., 2021), with similar recoveries for CBZ and even better ones for ENA and SIL, and therefore lower MDL and MQL. The measurement of the pharmaceuticals in the testing waters showed that the concentrations markedly dropped between the 48-h renewal time. Consequently, the exposure concentrations and derived empirical bioconcentration factors were calculated based on the average of the measured concentrations. The observed decreases in the water concentrations could be partially due to adsorption to the testing chamber walls (i.e., C _w 0_) and partially due to the absorption by the fish during the time between renewals. These results remark the need to measure the testing water concentrations and, if possible, use flow-through aquaria or reduce the time between renewals.

It is important to remark that no previous studies addressing the bioconcentration of ENA and SIL in fish under laboratory conditions are available in the international literature. The average measured concentrations in the whole body and BCF_m_ of pharmaceuticals studied in *C. decemmaculatus* after 96 h of exposure, showed the order CBZ ∼ SIL > ENA. The log Kow values of these drugs in decreasing order are 3.18 for SIL, 2.77 for CBZ, and 2.45 for ENA (Gobry et al., 2000; Loftsson et al., 2010; Huerta et al., 2013b), and seem not to explain the bioaccumulation extent of each pharmaceutical. Additionally, the pKa values are 6.78 and 9.12 for SIL, 2.77 and 13.9 for CBZ, and 3.0 and 5.5 for ENA (Gobry et al., 2000; Loftsson et al., 2010), so while SIL and CBZ are mainly neutral at tested pH, the ENA is found dominantly as a monovalent anion. In addition, the log Dow at the pH reported was 2.77 for SIL, 1.12 for CBZ, and 0.61 for ENA, showing that SIL and CBZ were moderate hydrophilic at the conditions of the test, while ENA with the lowest value was highly hydrophilic. This difference could explain the lower bioaccumulation of ENA as a reduction in bioavailability. However, it could be better comprehended as a quick metabolization of ENA. In mammals, the prodrug (ENA) is metabolized to the active drug, enalaprilat, through esterases. While in the rhesus monkey, dog, and man, this esterase activity is found in the liver, in rats, this is also in the plasma (Ulm, 1983). In this study, waterborne exposure to ENA was tested, so results suggest that, in fish, esterase activity would be present in the plasma and could quickly metabolize the drug, avoiding bioaccumulation.

The bioconcentration of CBZ in fish has also been studied in other fish species. In *Carassius carassius*, exposed during 7 d, the BCF_e_ was within 2.8 and 9.2 and the BCF_m_ 3.8 and 7.6 for the muscle and the liver, respectively, indicating that the plateau was almost reached in the tested time (Nkoom et al., 2020). In another study, in which the fish were exposed during 42 d, the BCF_m_ in the muscle and the liver were 1.9 and 4.6 for *Pimephales notatus* and 1.8 and 1.5 for *Ictalurus punctatus* (Garcia et al., 2012). Only one study has been found assessing the CBZ bioconcentration in a neotropical fish species. The BCF for *J. multidentata* after 48 h of exposure was 6 and 9 for the muscle and the liver, respectively (Valdés et al., 2016). The BCFs reported by these authors were all in the same order of magnitude as the one found in the present study, indicating a low bioconcentration capacity of this drug in fish.

The applied bioconcentration kinetic model for the SIL fit experimental data satisfactorily. Particularly for the *P. mesopotamicus* trial, a latency period term needed to be added. The four-day delay in *P. mesopotamicus* was one of the more evident differences among species. Also, the maximum internal concentrations and BCF_m_ values estimated by the model were unlike among species. Good regression (slope ≈ 1) has been reported between whole fish and muscle concentrations for hydrophobic non-ionized organic pollutants (Bevelhimer et al., 1997), but this relationship is not available for pharmaceuticals. Therefore several different factors including differential accumulation among tissues, biotransformation through the liver or gills, size species differences, analyzed tissue, the actual exposure concentration, latency time, tissue-specific uptake, and differences in the rates or mechanisms of uptake and depuration between fish species could explain the differences observed (Gomez et al., 2010; Tanoue et al., 2015; McCallum et al., 2017; McCallum et al., 2019). Especially the unlike size of each species and the different assessed body compartments could play a relevant role in this case. Individuals of *P. mesopotamicus* were more than 100 times weightier than *C. decemmaculatus*. Additionally, while in *P mesopotamicus*, only the muscle was analyzed, in *C. decemmaculatus*, the whole body was processed. It is known that body size affects contaminant bioaccumulation in fish (Sijm and van der Linde, 1995), but no previous studies were found assessing its effects on the toxicokinetics of pharmaceuticals. Results found here suggest that the time-to-distribution of SIL would increase with fish size. Moreover, the accumulation of pharmaceuticals, such as CBZ and diclofenac (Nkoom et al., 2020), in the muscle usually is slower and less extended than in other body compartments (i.e., the liver).

The higher bioconcentration estimated for *C. decemmaculatus* could also be linked with the lower elimination rate constant estimated for this species during the uptake phase (k_2u_), which was more than 40 times lower than the one estimated for *P. mesopotamicus*. A lower k_2_ value would reflect both a reduced capacity of depuration and/or biotransformation of SIL in *C. decemmaculatus* during the exposure period. On the other hand, the uptake rate constants (k_1_) were similar, as well as the elimination rate constants (k_2d_) and half-live (t_1/2_) estimated in the depuration phase. Particularly, the low k_1_ and k_2u_ values were consistent with the behavior of hydrophilic compounds, in which diffusion through membranes might be hindered by de pH of the media (Brox et al., 2016; Chen et al., 2017).

A notable outcome of this study was the difference between the BCF obtained for SIL in the 96 h and 7 d *C. decemmaculatus* trials. The higher BCF_e_ and BCF_m_ of the kinetic trial would indicate that longer exposure times are necessary for reaching the steady state of SIL bioconcentration. Although the BCF_m_ could be overestimated, results would point out a higher bioconcentration capacity for SIL concerning other drugs, encouraging further studies to obtain a more precise value. Remarkably, no information is still available regarding the BCF in fish for this drug.

Almost no studies are available assessing SIL bioaccumulation in feral fish. In a previous study, it was observed that maximum SIL bioaccumulation in the muscle of four species from the Rio de la Plata was relatively similar, varying between 29.03 and 71.7 μg/kg (Rojo et al., 2021). When those concentrations are compared with the accumulation level in the muscle of *P. mesopotamicus* in the lab, it is found that bioconcentration in the lab, in 1 week and without reaching the plateau, was more than 3-fold higher than the maximum bioaccumulation level observed in the field. This result would indicate that concentrations at which fish were exposed in the field were lower than those tested in the present study. That is consistent with the concentrations (12.8–560.9 ng/L) previously found in the surface waters of Argentina (Perez et al., 2018). Together, these findings let us hypothesize that waterborne exposure would be the main uptake pathway of SIL for fish.

International bioconcentration criteria established by the Canadian Government, the European Commission, the United States Environmental Protection Agency (USEPA), and the Stockholm Convention on Persistent Organic Pollutants consider “bioaccumulative” or “very bioaccumulative” substances with BCF values between 1,000 and 5,000. According to the BCF values obtained for CBZ, ENA, and SIL in *C. decemmaculatus* after 96 h exposure and for SIL in *P. mesopotamicus* after 7 d exposure in the kinetic trial, well below 1,000, these compounds would be classified in the category of low bioconcentration potential. The exception was the BCF_m_ estimated for SIL in C. *decemmaculatus*, with a value of 3,143. However, that values would need to be confirmed in further trials considering a longer exposure time.

## 5 Conclusion

The BCFs for CBZ, ENA, and SIL were measured for the first time in the neotropical fish species, being for CBZ and SIL 1.5 and 1.4, respectively, and for ENA two orders of magnitude lower (0.007), possibly because of its biotransformation in enalaprilat. Obtained BCF values were like those reported for frequently studied holarctic fish species and range among the ones for compounds classified in the category of low bioconcentration potential.

The bioconcentration kinetics of SIL was evaluated for the first time in fish, comparing the process in two native species. Not only differences were observed among species, but also in comparison with the other tested drugs. Bioconcentration of SIL was faster and higher in the whole body of *C. decemmaculatus*, the smaller fish than in the muscle of *P. mesopotamicus*, the bigger one. Compared with the other assessed pharmaceuticals, SIL bioconcentration kinetics showed a longer time to reach the plateau and at least one order of magnitude higher BCF. Further studies assessing longer exposure times than 1 week are encouraged for precisely estimating its BCF.

Finally, the study contributes new information about the bioconcentration of three frequently detected pharmaceuticals in the Del Plata Basin for poorly studied neotropical fish species, which results in helpful inputs for future ecological risk assessments.

## Data Availability

The raw data supporting the conclusion of this article will be made available by the authors, without undue reservation.
