# Supplementary material for: Bioconcentration of carbamazepine, enalapril, and sildenafil in neotropical fish species
Source: Front Toxicol. 2023 Oct 3;5:1247453. doi: 10.3389/ftox.2023.1247453 (PMC10579815; doi:10.3389/ftox.2023.1247453)
Supplement: Supplementary file 1 [file Table1.DOCX]

| **Table S1. Precursor ions, product ions, and retention times (RT) of compounds studied** | | | |
| --- | --- | --- | --- |
| **Compound** | **Precursor ion (m/z)** | **Product ion**  **(m/z)** | **RT (min)** |
| Carbamazepine | 237 [M+H]^+^ | 194 | 7.17 |
| Enalapril | 377 [M+H]^+^ | 355 | 5.51 |
| Sildenafil | 475 [M+H]^+^ | 237 | 5.29 |
