# Supplementary material for: Bioconcentration of carbamazepine, enalapril, and sildenafil in neotropical fish species
Source: Front Toxicol. 2023 Oct 3;5:1247453. doi: 10.3389/ftox.2023.1247453 (PMC10579815; doi:10.3389/ftox.2023.1247453)
Supplement: Supplementary file 2 [file Table2.DOCX]

| **Table S.2** CBZ, ENA, and SIL water concentration | | | |  |
| --- | --- | --- | --- | --- |
| **Compound** | **C_w o_** | **C_w 96 h_** |  |  |
| Carbamazepine | 226 | 44.4 |  |  |
| Enalapril | 573 | 45.3 |  |  |
| Sildenafil | 128 | 12.7 |  |  |
| C_w o_ concentration in water at 0 h (µg/L) C_w 96 h_: concentration in water at 96 h (µg/L) | | | | |
