# Supplementary material for: Bioconcentration of carbamazepine, enalapril, and sildenafil in neotropical fish species
Source: Front Toxicol. 2023 Oct 3;5:1247453. doi: 10.3389/ftox.2023.1247453 (PMC10579815; doi:10.3389/ftox.2023.1247453)
Supplement: Supplementary file 3 [file Table3.DOCX]

| **Table S3**. Concentration of SIL measured in *C. decemmaculatus* whole body | | | | | | | | |
| --- | --- | --- | --- | --- | --- | --- | --- | --- |
| Phase | t (d) | C_t_ (av) |  | SE | n | C_w (o)_ | C_w (f)_ | |
| Uptake | 0 | <MDL |  |  | 2 | 81.6 | 1.28 | |
|  | 1 | 482 | ± | 54.3 | 2 |  |  | |
|  | 3 | 593 | ± | 25.4 | 3 |  |  | |
|  | 5 | 806 | ± | 83.4 | 3 |  |  | |
|  | 7 | 1769 | ± | 106.3 | 3 |  |  | |
| Depuration | 9 | 724 | ± | 226 | 3 |  |  | |
|  | 11 | 221 | ± | 3.04 | 2 |  |  | |
| t: exposure time (days), C_t (av)_: average concentration in fish tissue (whole body) (µg/kg), SE: standard error, n: number of samples, C_w o_: average concentration in water after standard addition (µg/L); C_w f_: average concentration in water before renewals (11 d) (µg/L) | | | | | | | |  |
