# Supplementary material for: Bioconcentration of carbamazepine, enalapril, and sildenafil in neotropical fish species
Source: Front Toxicol. 2023 Oct 3;5:1247453. doi: 10.3389/ftox.2023.1247453 (PMC10579815; doi:10.3389/ftox.2023.1247453)
Supplement: Supplementary file 4 [file Table4.DOCX]

| **Table S4.** Concentration of SIL measured in *P. mesopotamicus* muscle | | | | | | | |
| --- | --- | --- | --- | --- | --- | --- | --- |
| Phase | t | C_t (av)_ |  | SE | n | C_w o_ | C_w f_ |
| Uptake | 0 | <MDL |  |  | 6 | 32.3 | 0.15 |
|  | 2 | 0.37 | ± | 0.1 | 8 |  |  |
|  | 4 | 25 | ± | 7.2 | 7 |  |  |
|  | 6 | 241 | ± | 43.4 | 8 |  |  |
| Depuration | 8 | 102 | ± | 13.8 | 9 |  |  |
|  | 10 | 92 | ± | 5.6 | 8 |  |  |
| t: test time (days), C_t (av)_: average concentration in fish tissue (µg/kg), SE: standard error, n: number of samples with detected concentration, C_w o_: average concentration in water after standard addition (µg/L); C_w f_: average concentration in water before renewals (µg/L) | | | | | | | |
|  |  |  |  |  |  |  |  |
